# Antiproliferative and Antimicrobial Effects of *Rosmarinus officinalis* L. Loaded Liposomes

**DOI:** 10.3390/molecules27133988

**Published:** 2022-06-21

**Authors:** Irina Ielciu, Mihaela Niculae, Emoke Pall, Cristina Barbălată, Ioan Tomuţă, Neli-Kinga Olah, Ramona Flavia Burtescu, Daniela Benedec, Ilioara Oniga, Daniela Hanganu

**Affiliations:** 1 Department of Pharmaceutical Botany, Faculty of Pharmacy, “Iuliu Haţieganu” University of Medicine and Pharmacy Cluj-Napoca, 400010 Cluj-Napoca, Romania; 2 Department of Clinical Sciences, University of Agricultural Sciences and Veterinary Medicine Cluj-Napoca, 400374 Cluj-Napoca, Romania; mihaela.niculae@usamvcluj.ro (M.N.); emoke.pall@usamvcluj.ro (E.P.); 3 Department of Pharmaceutical Technology and Biopharmaceutics, Faculty of Pharmacy, “Iuliu Haţieganu” University of Medicine and Pharmacy Cluj-Napoca, 400010 Cluj-Napoca, Romania; tomutaioan@umfcluj.ro; 4 PlantExtrakt Ltd., Rădaia, 407059 Cluj-Napoca, Romania or olah.neli@uvvg.ro (N.-K.O.); ramona.burtescu@plantextrakt.ro (R.F.B.); 5 Department of Medicinal Chemistry and Pharmaceutical Industry, Faculty of Pharmacy, “Vasile Goldiş” Western University of Arad, 310414 Arad, Romania; 6 Department of Pharmacognosy, Faculty of Pharmacy, “Iuliu Haţieganu” University of Medicine and Pharmacy Cluj-Napoca, 400010 Cluj-Napoca, Romania; dbenedec@umfcluj.ro (D.B.); ioniga@umfcluj.ro (I.O.); dhanganu@umfcluj.ro (D.H.)

**Keywords:** *R. officinalis*, liposomes, polyphenols, antiproliferative, adenocarcinoma, hepatic cells, antimicrobial

## Abstract

*Rosmarinus officinalis* L. is a species that is widely known for its culinary and medicinal uses. The purpose of the present study consisted of the evaluation of the antiproliferative and antimicrobial effects of *R. officinalis*-loaded liposomes (L-R). Characterization of the liposomes was performed by establishing specific parameters. The load of the obtained liposomes was analyzed using an LC-MS method, and antiproliferative assays evaluated the cell viability on a liver adenocarcinoma cell line and on a human hepatic stellate cell line. Antimicrobial assays were performed by agar–well diffusion and by broth microdilution assays. The obtained liposomes showed high encapsulation efficiency, suitable particle size, and good stability. High amounts of caffeic (81.07 ± 0.76), chlorogenic (14.10 ± 0.12), carnosic (20.03 ± 0.16), rosmarinic (39.81 ± 0.35), and ellagic (880.02 ± 0.14) acids were found in their composition, together with other polyphenols. Viability and apoptosis assays showed an intense effect on the cancerous cell line and a totally different pattern on the normal cells, indicating a selective toxicity towards the cancerous ones and an anti-proliferative mechanism. Antimicrobial potential was noticed against all tested bacteria, with a better efficacy towards Gram-positive species. These results further confirm the biological activities of *R. officinalis* leaf extract, and proposes and characterizes novel delivery systems for their encapsulation, enhancing the biological activities of polyphenols, and overcoming their limitations.

## 1. Introduction

*Rosmarinus officinalis* L. (rosemary) is a species belonging to the Lamiaceae family, which is known for its medicinal and culinary uses. It is widespread in the Mediterranean region, where it was first identified in the temperate climate [1,2]. It is an evergreen shrub, with linear, sessile, needle-like silver leaves and purple–blue bilabiate flowers grouped in lax inflorescences [1,3,4,5,6]. It is cultivated worldwide and has been used in folk medicine for the treatment of muscle spasms, renal colics, and dysmenorrhea [7]. The vegetal medicinal product is provided by the leaves [1,8,9], which contain significant amounts of essential oils that are mixtures of volatile compounds, such as monoterpenes, sesquiterpenes, and aromatic compounds, with camphor, 1,8-cineole, α-pinene, borneol, camphene, β-pinene, and limonene being the majority compounds [1,4,10,11]. Together with the essential oil, the phenolic compounds are also found in significant amounts in the rosemary chemical composition, with rosmarinic acid, caffeic acid, ursolic acid, betulinic acid, carnosic acid, and carnosol being the most common ones [1,4,9,12]. The biological activities these compounds exhibit are various and concern the antibacterial, antifungal, anti-inflammatory, anti-proliferative, hepatoprotective, antinociceptive and antidiabetic properties [7,8,9,13]. Therefore, vegetal medicinal products belonging to this species are used to treat conditions that are related to the nervous, cardiovascular, gastrointestinal, genitourinary, menstrual, hepatic, and respiratory systems [7]. The species presents a monograph that was released by the European Medicines Agency (EMA) in 2010 [14], which describes the use of leaves and essential oils as drugs [8]. It is a species that is known for its antioxidant properties, which represents the basis or has important synergistic effects with numerous other biological activities, such as the antimicrobial [15,16] or the cytotoxic ones [17,18,19]. These activities may be assigned to their essential oil composition [16,20], and especially to polyphenols, which are found in significant amounts in the composition of rosemary [1,4,5,8,21]. The phenolic compounds that are responsible for the biological activities of *R. officinalis* belong to the class of phenolic diterpenes (carnosic acid and carnosol) and to the class of phenolic acids (rosmarinic acid) [4,22]. They are responsible for the antimicrobial, antioxidant, antiproliferative, hepatoprotective, and antihyperglycemic activities [2,4,10,22,23].

The phenolic compounds’ role in different biological activities are limited due to low oral bioavailability in relationship with the chemical structure, molecular size, degree of polymerization, and water solubility, and these limitations may be exceeded by their incorporation into different drug-delivery systems, of which liposomes seem to be the most adequate [24].

Nano-phytomedicines represent a category of drug formulations that result from a combination of nanotechnology and herbal medicine [25]. They are modern formulations that are meant to overcome the disadvantages of vegetal medicinal products or the extracts that are obtained on their basis, which present promising in vivo and in vitro biological activities, but show at the same time significant limitations, such as poor absorption, low bioavailability, low solubility, and rapid clearance [25,26,27]. Nanotechnology has proven to be an effective tool that can be used to effectively eradicate these limitations [25,26]. Liposomes are artificial vesicles of small size and spherical shape, obtained from cholesterol and non-toxic phospholipids [27,28]. Such preparations exist in the case of *R. officinalis* and are used to achieve more efficacy in overcoming the limitations of its phytoformulations (related to bioavailability and solubility) and to enhance its therapeutical activities [29,30]. Liposomes have also proven to be effective in the encapsulation of the essential oil obtained from rosemary [21,29,30]. At the same time, these formulations have also proven to improve the release of *R. officinalis* extract [23] or to encapsulate its main compounds, especially polyphenols, by enhancing their biological potential and limiting their major disadvantages [31]. Through the encapsulation of *R. officinalis* extract in liposomal carriers, it was proven that these formulations may improve the limitation of using polyphenols. Existing studies cite better penetration enhancement for topical delivery [23], a more sustained release [20], an increased bioavailability [30], or the enhancement of the biological activities [15], compared to conventional extract systems.

With all of this taken into consideration, the aim of the present study consisted of the investigation of the encapsulation into liposomes of an *R. officinalis* leaf extract and testing its antimicrobial and antiproliferative potential. The novelty of the present study consists of the fact that, to the best of our knowledge, it is the only study that proposes and characterizes special delivery systems such as liposomes for the encapsulation of *R. officinalis* leaf extract, with the final purpose of enhancing the biological activities of its polyphenols, such as the antimicrobial and cytotoxic ones. The developed nanoformulations may represent innovative and specific vectors for the amelioration of the limitations of polyphenols, preserving or enhancing the biological properties of *R. officinalis* leaf extracts.

## 2. Results

### 2.1. Preparation and Characterization of Liposomes

The results obtained for the characterization of empty liposomes (L-E), those loaded with doxorubicin (L-DOX), and the R. officinalis leaf extract (L-R) (Table 1) highlight the fact that all three liposomal formulations were monodisperse, with suitable particle size and a high encapsulation efficiency (EE%). The negative Zeta potential values suggest a good stability of the liposomal dispersions.

### 2.2. Phytochemical Analysis

LC-MS analysis of L-R showed significant amounts of polyphenols (Table 2, Figures S1 and S2). The compounds that were found in the highest amounts belong to the class of phenolic acids (caffeic acid, chlorogenic acid, rosmarinic acid, and ellagic acid). Carnosic acid, a diterpenic acid, and carnosol, its corresponding phenolic diterpene, were also found in significant amounts. However, ellagic acid (the dimerization compound of gallic acid) was found to be the majority compound in the composition of the tested liposomes, followed by caffeic acid and chlorogenic acid. Flavonoids that were found in the composition of the liposomes were represented both by aglycons (apigenin, luteolin, chrysin, quercetin, naringenin, hesperetin) and by glycosides (luteolin-7-O-glucoside, rutoside, hyperoside).

### 2.3. Cytotoxicity Assays

**Figure 1:**
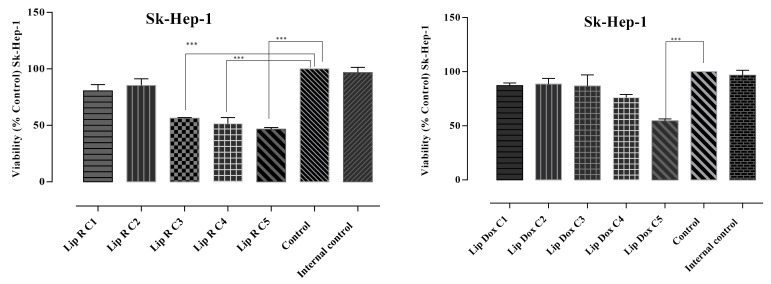
Antiproliferative potential towards SK-Hep-1 cell line induced by five different concentrations of L-R, calculated according to the TPC (µmol GAE/mL) determined for each extract (56.6 µM, 113 µM, 169.5 µM, 226 µM, and 282.5 µM) and five different concentrations of L-DOX C1–C5 (1.64 µM, 8.2 µM, 12.3 µM, 16.4 µM, and 20.5 µM). Negative control—untreated cells, internal control—L-E. Values are represented as the mean of viability ± SD of three determinations. *** *p* < 0.0001 (differences between extract-treated cells and negative control, L-R versus negative control).

**Figure 2:**
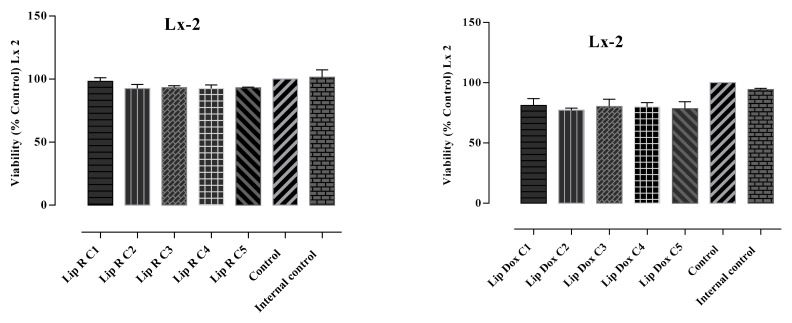
Antiproliferative potential of L-R and L-DOX on LX-2 cell line tested at five different concentrations of L-R calculated according to the TPC (µmol GAE/mL) determined for each extract (56.6 µM, 113 µM, 169.5 µM, 226 µM, and 282.5 µM) and doxorubicin C1–C5 (1.64 µM, 8.2 µM, 12.3 µM, 16.4 µM, and 20.5 µM). Negative control—untreated cells, internal control—L-E. Values represent the mean ± SD of three determinations.

The in vitro antiproliferative potential of the L-R and L-DOX was assessed on two selected human cell lines, SK-Hep-1 (HTB-52^™^), derived from the ascitic fluid of a patient with adenocarcinoma of the liver, and LX-2, a human hepatic stellate cell line. The MTT assay was performed to evaluate cytotoxicity. Results were correlated with the ones obtained in the apoptosis assays. The viability and apoptosis assays showed different behavior of normal and tumoral cells exposed to the loaded liposomes. The performed analysis indicated relevant in vitro cytotoxic activity for all tested samples as well as differences related to doses and cell lines. The obtained results are presented in Figure 1 and Figure 2.

The results pointed out relevant in vitro cytotoxic activity expressed by L-R against the SK-Hep-1 (HTB-52™), with the most intense antiproliferative effect observed in the case of the highest tested concentration—C5 (282.5 µM GAE/mL). The concentration of L-R that required the SK-Hep 1 cell viability to be reduced by 50% (IC50) was 181.29 ± 6.14 GAE/mL.

**Figure 3:**
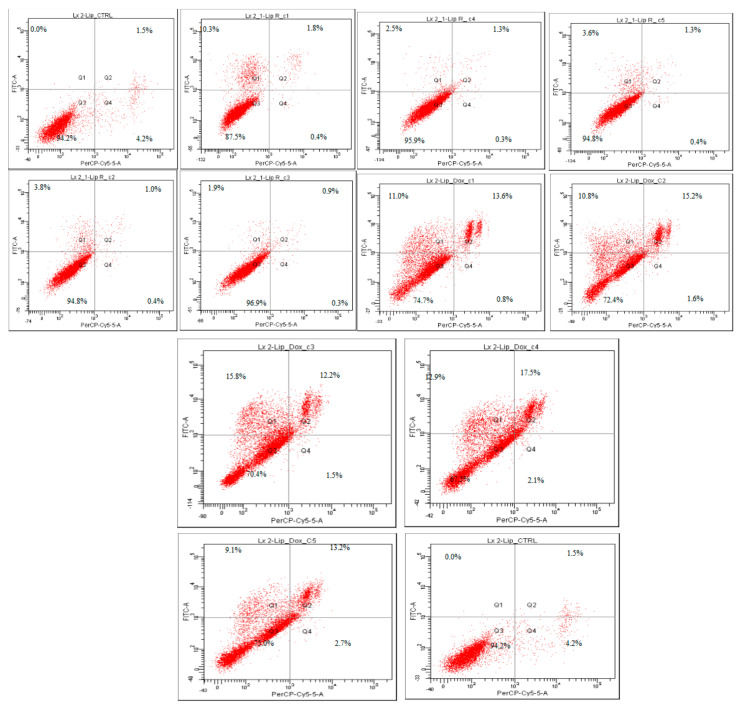
Apoptosis evaluation in LX2 cells (10,000 events) after treatment with L-R and L-DOX. After 24 h of treatment, the cells were stained with Annexin V-FITC and Ethidium Homodimer III (EthD-III) and were evaluated using the BD FACS Canto II flow cytometer (Becton Dickinson, Franklin Lakes, NJ, USA) using the BD FACSDiva 6.1.2 software. The results are shown in dot plots divided into 4 quadrants: Q3—viable cells (Annexin V-FITC (−), EthD-III (−)), Q1—early apoptotic cells (Annexin V-FITC (+), EthD-III (−)), Q2—late apoptotic (Annexin V-FITC (+), EthD-III (+)), and Q4—necrotic cells (Annexin V-FITC (−), EthD-III (+)).

**Figure 4:**
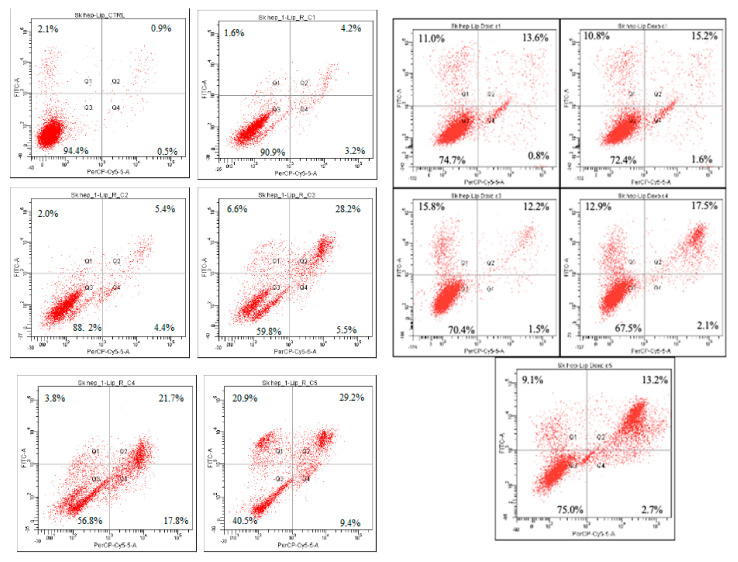
Apoptosis evaluation in SK-Hep-1 (HTB-52^™^) (10,000 events) after treatment with L-R and L-DOX. After 24 h of treatment, the cells were stained with Annexin V-FITC and Ethidium Homodimer III (EthD-III) and were evaluated using a BD FACS Canto II flow cytometer (Becton Dickinson, USA) using the BD FACSDiva 6.1.2 software. The results are shown in dot plots divided into 4 quadrants: Q3—viable cells, (Annexin V-FITC (−), EthD-III (−)), Q1—early apoptotic cells (Annexin V-FITC (+), EthD-III (−)), Q2—late apoptotic (Annexin V-FITC (+), EthD-III (+)), and Q4—necrotic cells (Annexin V-FITC (−), EthD-III (+)).

The SK-Hep-1 (HTB-52^™^) and LX- 2 cells were exposed to different concentrations of L-R and L-DOX for 24 h and apoptosis was measured by the Annexin V-FITC and Ethidium homodimer III assay. The results are shown in Table 3 and Figure 3 and Figure 4.

Apoptosis assay showed that the treatment with L-R and L-DOX induced apoptosis and necrosis at varying rates in SK-Hep 1 and LX-2 cells compared to the control. The pattern of cell inhibition was different in the two cell lines and depended on the concentration of tested substance. The addition of L-R (C1-C5) to the SK-Hep 1 cell culture induced cellular death effect following 24 h compared to untreated cells. The percentages of the viable cells induced by L-R C3, C4, and C5 (59.8, 56.8, and 40.5, respectively) were similar to those determined by the highest tested concentration of doxorubicin, the positive control. The cell death mechanism was predominantly apoptosis, but in C4 it was also necrosis, with the highest percentage of necrotic cells (17.8%). After treatment with C4, the apoptotic population was 3.8%, followed by a significant increase in apoptosis rate, correlated with the concentration of L-R. Compared with other concentrations, the cells treated with C5 presented the highest rates of apoptosis, at 20.9% and 29.2%, respectively, and cell necrosis was 9.45%.

The pattern of cellular inhibition obtained in the case of L-DOX was different compared to L-R. The percentage of cell viability was slightly decreased, but was still maintained at over 80% in C1–C3, with a higher decrease in C4 (74.2%) and especially in C5, where the percentage of cell viability was 46.3% and the apoptotic cells population was 47.7%. All data are correlated with the results obtained in the MTT test, in which the same pattern of cell viability decrease was noticed. The L-R cytotoxicity was noticed only towards the tumoral cell line, whereas on the normal cell line (human hepatic stellate cell line, LX-2) the percentages of viable cells was within the range. Although the same liposomes did not exhibit a similar effect in the LX-2 cell line, L-R-induced apoptosis rates were significantly lower on the LX-2 cells, demonstrating selective toxicity of L-R. Regarding the effect of L-DOX in LX-2 cells, the average rates of apoptosis recorded was 26.42 ± 2.73%. To enhance apoptotic level, co-delivery of Doxorubicin and *R. officinalis* may be a fairly viable alternative.

### 2.4. Antimicrobial Activity Assays

Results of the in vitro antimicrobial activity evaluation are presented in Table 4 and Table 5 and Figure S2.

The tested product displayed in vitro antimicrobial activity (Table 4) against all selected bacterial reference strains. Based on the values of the inhibition zone diameter, the highest effect was recorded towards the Gram-positive species (*Bacillus cereus* > *MSSA* > MRSA > *Enterococcus faecalis*) compared to the Gram-negative ones (*Salmonella enterica* serovar Enteritidis = *Salmonella enterica* serovar Typhimurium > *Escherichia coli*). Compared to the positive control gentamicin, these values were found to be significantly (*p* < 0.05) higher in case of *Bacillus cereus*, MSSA, MRSA, and *Enterococcus faecalis*. Against *Enterococcus faecalis* and the selected Gram-negative bacteria, L-R presented inhibitory activity similar to that of gentamicin (*p* > 0.05).

Similarly, the minimum inhibitory and bactericidal concentrations established using the broth microdilution method indicated better antimicrobial activity against the Gram-positive strains (Table 5). Considering the MIC index, the tested product manifested bactericidal activity towards all tested bacterial species (MBC/MIC ≤ 4).

## 3. Discussion

*R. officinalis* is one of the most widely known medicinal species due to its use in the treatment of numerous pathologies. The vegetal medicinal product obtained from this species is provided by the leaves, which contain large amounts of essential oils and polyphenols. The present study aimed to bring novelty insight concerning this species by proposing novel formulations as special delivery systems. These formulations were represented by liposomes, encapsulating the leaf extract of the species, with the final purpose of overcoming the limitations of polyphenol administration.

One of the most important advantages of liposomes is that they can incorporate both lipophilic and hydrophilic compounds, making them suitable candidates for the encapsulation and delivery of plant extracts [32]. Yet, achieving the desired in vitro/in vivo effect is dependent on the quality attributes of the liposomal formulation, i.e., the encapsulated drug concentration, liposomal size, polydispersity index, or zeta potential [33,34]. The characterization of the proposed liposomal formulations (Table 1) evidenced the achievement of good delivery systems that are in agreement with previous reports [35,36], recommending them for the assessment of polyphenolic content and their antiproliferative and antimicrobial potentials. Other similar studies were based on encapsulating rosemary essential oil [16,20,21,30], but also on its extracts [23,36,37,38]. To the best of our knowledge, the present study is among the few that describe the encapsulation of a leaf extract of the species, representing the only study that describes the obtention of special delivery systems aimed at enhancing the antimicrobial and antiproliferative potentials of polyphenols from its derived vegetal medicinal product. Most of the existing studies are focused on the antioxidant [16,20,36] or antifungal activities [31] of these delivery systems.

Phytochemical analysis of liposomes showed significant amounts of phenolic acids, with high contents of ellagic, rosmarinic, caffeic, and chlorogenic acids. Carnosic acid and carnosol were the majority diterpenic phenols, and flavonoids were represented by apigenin, chrysin, rutoside, hyperoside, and luteolin-7-O-glucoside. The obtained results provide further arguments that confirm the presence of these compounds in the composition of the species, especially rosmarinic, caffeic, and chlorogenic acids, which were previously identified [1,9,12,23,39]. Carnosic and carnosol were also previously identified as majority compounds in the leaves of rosemary [9,12,21,23,39,40]. Among flavonoids, kaempferol, luteolin, and their glucosides were also previously identified in the leaves of the species [9,12]. Hesperetin was also previously identified by our group of authors, together with other compounds, but in the composition of young shoots [13]. The obtained liposomes proved therefore to maintain the compounds that were identified previously in the composition of the vegetal material. The most innovative aspect of the present study is therefore the high amount of ellagic acid that was found, together with significant amounts of rutoside and hyperoside (Table 2), in the composition of the liposomes. All these identified and quantified compounds provide further knowledge on the species and confirming its chemical composition.

Regarding the antiproliferative assays, to the best of our knowledge, the results obtained in the present study for the antiproliferative assays are the first from a liver adenocarcinoma cell line (SK-Hep-1 (HTB-52^™^), a cell line derived from the ascitic fluid of a patient with adenocarcinoma of the liver) and on a human hepatic stellate cell line (LX-2), as there are no previous studies concerning liposomes loaded with rosemary-leaf extract on the two cell lines selected for the study. The choice of the two cell lines is directly related to previous reports on hepatoprotection performed by our team. This represents one of the most important aspects of novelty of the present study, together with the obtention of liposomes as special delivery systems for polyphenols. Moreover, the present study represents the first to test the antiproliferative potential of these delivery systems. The cytotoxicity and apoptosis analysis showed a different comportment of normal and tumor cells treated with L-R and L-DOX. The treatment of SK-Hep-1 cells to L-R induced a significant decrease in cell viability, and this decrease was influenced by the concentration of loaded samples (up to 169.6 µM GAE/mL). The reduction of cell viability was dose dependent; the lowest viability was identified in cells treated with the highest concentration of natural extract (C5), where the mean viability was 46.68 ± 1.32%. Similar behavior was also observed in cells treated with L-DOX, stating that the decrease in cell viability was not as high (mean viability—54.44 ± 1.92%). According to the apoptosis assay results, the decreased viability was associated with increased percentages of apoptotic and necrotic cells. The predominant mechanism of cell death induced by L-R extract in Sk-Hep-1 was therefore related to apoptosis. The cytotoxic potential was also assessed by calculating the required concentration that inhibited 50% of the Sk-Hep-1 cell line (IC_50_ in µM/mL). The concentration of L-R required to reduce Sk-Hep-1 cell viability by 50% was 181.29 ± 6.14 μM/mL at 24 h. For L-DOX IC_50_ it was 10.59 ± 0,40 µM/mL. The mechanism of protection still needs further investigation, but offers a good background for novel insights.

Several studies documented the ability of distinct *R. officinalis*-derived products (essential oils and alcoholic, acidic, aqueous extracts) to inhibit bacterial strains [21,41,42]. Ethanolic extracts are listed among the most potent, in particular when tested against Gram-positive bacteria. Nevertheless, few studies have performed assays on liposomes, with most of them concentrating on the encapsulation of its essential oils [16,20] and very few on its polyphenols [31,38]. The present study provides novel insight into the antimicrobial activity of this species by assessing the antimicrobial activity of rosemary-leaf extract-loaded liposomes and attributing this activity to polyphenols. The results of this study are, however, in accordance with previous reports that indicated a more intense antimicrobial effect against MSSA, MRSA, *Bacillus cereus*, and *Enterococcus faecalis*. A relevant finding of this study is the inhibitory and bactericidal activity displayed against the MRSA reference strain. MRSA is recognized worldwide as a well-known prototype of multidrug resistance, and alternatives to classical antimicrobial agents are needed [43]. L-R demonstrated significant anti-MSSA and -MRSA activity in vitro, whereas in case of the three Gram-negative strains, the results were comparable to those obtained for the positive control, gentamicin. The lower intrinsic susceptibility commonly described in the case of Gram-negative bacteria is explained based on the composition and structure of the bacterial wall. On the other hand, the antibacterial activity of plant-originating antimicrobial compounds or products is ensured by mechanisms such as the bacterial cell membrane, the inhibition of efflux pumps, and/or DNA and protein biosynthesis [44]. Furthermore, the type of action is relevant, and the tested product L-R was found to have bactericidal potential against both Gram-positive and Gram-negative bacteria.

## 4. Materials and Methods

### 4.1. Chemicals and Reagents

Egg phospholipids with 80% phosphatidylcholine (Lipoid E80) and 1,2-dipalmitoyl-sn-glycero-3-phosphocholine (DPPC) were purchased from Lipoid GmbH (Ludwigshafen, Germany), cholesterol (CHO) from sheep wool, and doxorubicin hydrochloride (DOX) and Folin-Ciocalteu reagent from Merck KgaA (Darmstadt, Germany). Minimum Essential Medium Eagle (MEM), Dulbecco’s modified Eagle’s medium (DMEM, High glucose), Antibiotic-Antimycotic 100×, and 1% L-glutamine were purchased from Merck KgaA (Darmstadt, Germany), and 10% fetal bovine serum from EuroClone (MI, Pero, Italy). 3-(4,5-dimethylthiazol-2-yl)-2,5-diphenyl tetrazolium bromide was purchased from Merck KgaA (Darmstadt, Germany), and dimethyl sulfoxide solution (DMSO) from Fluka (Buchs, Switzerland). Bacterial reference strains were obtained from Oxoid Ltd. (Hampshire, UK), whereas the culture mediums, Mueller Hinton Broth and Mueller Hinton agar, were purchased from Merck (Darmstadt, Germany). All the other solvents and reagents used for analysis were of analytic-grade purity and were purchased from Merck KgaA (Darmstadt, Germany). Reference compounds used in the LS-MS method were also of analytic-grade purity and were purchased from Phytolab, (Vestenbergsgreuth, Germany).

### 4.2. Preparation of Extracts

The vegetal material consisted of the leaves of *Rosmarinus officinalis* obtained from the ecological culture of PlantExtrakt (Rădaia, Cluj county, Romania, latitude 46°48′05.54′′ N, 23°27′51.62′′ E). Vegetal material was identified by Lecturer Irina Ielciu, PhD, and voucher specimens were deposited at the herbarium of the Pharmaceutical Botany Department of the Faculty of Pharmacy Cluj-Napoca (Vouchers no. RO122.1-3). Air-dried leaves were ground using a Grindomix GM 200 knife mill (Éragny, France) and subsequently macerated for 10 days with 70% *v/v* ethanol in water and 2–3 shakes/day and filtered at the end of the maceration period [45]. For the obtained tincture, organoleptic properties, relative density, and residue at evaporation were assessed. Relative density was measured at 0.905 ± 0.002 using a Mettler Toledo (Greifensee, Switzerland) digital densimeter and the dry residue content was established at 2.65% ± 0.002 using a Kern thermoanalytical scale (Berlin, Germany) and a Memmert drying cabinet (Schwabach, Germany). Alcoholic content was measured at 65% ± 0.5 (by volume) [13]. The humidity was determined using the loss-on-drying method and Ohaus MB45 (USA) moisture balances. A quantity of approximately 1 g of product was dispersed on the sample moisture balance pan and dried at 100 °C. Loss on drying was automatically calculated at the moment when the weight variation was lower than 0.2% over 5 min. The analyses were performed in triplicate and the average was reported. Moisture content was established at 8.05% ± 0.68. After milling, the granulometric analysis of the vegetable product was performed by using a set of 9 sieves (Retsch, Haan, Germany) with sizes ranging between 100 and 900 µm. A quantity of approximately 50 g was separated by the sieves and the mean size and polydispersity index were calculated. Granulometric analysis reported results of 720 µm ± 14.23% [45].

### 4.3. Preparation of Liposomes

#### 4.3.1. Liposomes Loaded with *R. officinalis* Extract

The liposomes loaded with *R. officinalis* (L-R)-leaf extract were prepared using a modified method of the reverse-phase evaporation technique reported by Machado et al. [46]. Firstly, Lipoid E80 (70 mM) and CHO (7 mM) were dissolved in ethanol in a round-bottom flask. Then, *R. officinalis* extract (a volume 50% higher in relation to the final liposomal dispersion) and a specific amount of double-distilled water (calculated considering the water content of the plant extract) were added over the ethanolic solution. The final mixture was stirred for 5 min at 400 rpm, after which the ethanol was evaporated under pressure at 45 °C using the rotavapor. The liposomal dispersion was downsized using a water-bath sonicator for 15 min, and the purification step was performed via centrifugation at 5000 rpm for 15 min. The supernatant was collected and used in the subsequent experiments. The same technique was applied to prepare the empty liposomes (L-E) without adding the plant extract. Equal volumes of ethanol and double-distilled water were used to prepare the L-E.

#### 4.3.2. Liposomes Loaded with DOX

Liposomes loaded with DOX (L-DOX) were prepared using the active-loading method with ammonium sulfate [47,48]. To that end, the lipidic components, namely, DPPC (40 mM) and CHO (4 mM), were dissolved in ethanol in a round-bottom flask, after which the solvent was evaporated under pressure. The obtained lipid film was hydrated with ammonium sulfate solution (250 mM; pH = 5). The solvent evaporation and film hydration were performed using a rotavapor set to 45 °C. The multilamellar vesicles were downsized using a LiposoFast LF-50 (Avestin Europe GmbH, Mannheim, Germany) extruder and polycarbonate membranes with pore sizes of 800, 200, and 100 nm. The liposomal dispersion was purified against saline for 3 h using Slide-A-Lyzer dialysis cassettes with a molecular weight cut-off of 10 kDa, after which DOX was incubated with the liposomes for 15 min at 60 °C using the rotavapor. The L-DOX was purified against saline for 24 h at 4 °C using the dialysis cassettes mentioned above.

### 4.4. Characterization of Liposomes

#### 4.4.1. Total Polyphenolic Content (TPC)

The quantification of the polyphenolic content of L-R was performed using the colorimetric reaction with Folin–Ciocalteu reagent as reported by Postescu et al. [49]. In this respect, liposomes were diluted in methanol (1/10 *v/v/v*), and the solution was treated with Folin–Ciocalteu reagent diluted in water (1:10 *v*/*v*) and an aqueous solution of Na₂CO₃ 7.5%, according to the previously mentioned protocol. The absorbance of the supernatant was measured at 740 nm using a UV-VIS spectrophotometer (Specord 200 Plus, Analytik Jena, Jena, Germany).

#### 4.4.2. DOX Content

DOX encapsulated in liposomes was quantified using an HPLC-validated method with UV detection [35]. After diluting the liposomal dispersion in methanol 1:50 (*v*/*v*), the measurements were performed. The equipment used for DOX quantification was an Agilent 1100 Series HPLC system (Agilent Technologies, Santa Clara, CA, USA) and a Zorbax C18 column (3.5 μm) (Phenomenex, Torrance, CA, USA). The quantification method involved a gradient elution with acetonitrile and formic acid 0.1%. The DOX retention time was 0.95 min.

#### 4.4.3. Encapsulation Efficiency

The encapsulation efficiency (EE%) of the liposomes was calculated using the following formula:

$$\mathrm{EE}\%=\frac{\mathrm{Encapsulated}\;\mathrm{drug}\;\mathrm{concentration}}{\mathrm{Total}\;\mathrm{drug}\;\mathrm{concentration}}\times \;100$$

#### 4.4.4. Particle Size, Polydispersity Index, Zeta Potential

Particle size and polydispersity index (PdI) were determined by the dynamic light-scattering method, and zeta potential by laser Doppler electrophoresis using a Zetasizer NanoZS analyzer (Malvern Instruments Co., Malvern, UK). Prior to these measurements, the liposomes were diluted in double-distilled water 1:100 (*v*/*v*), and each sample was analyzed in triplicate.

### 4.5. LC/MS Analysis

Liposomes were sonicated for 30 min and then diluted 1 to 5 with HPLC-grade methanol. One μL of this diluted solution was subjected to HPLC analysis. Determinations were performed in triplicate and the quantitative data were statistically analyzed using the Microsoft Excel software. All quantitative determinations are expressed as mean ± RSD. Only the compounds under quantification limits (<LOQ) could be identified. Identified and quantified compounds can be found in Table 2.

#### LC/MS Apparatus

The LC/MS analysis was carried out on a Shimadzu Nexera I LC/MS-8045 (Kyoto, Japan) UHPLC system equipped with a quaternary pump, an autosampler, an ESI probe, and a Quadrupole rod mass spectrometer. Separation was obtained using a Luna C18 reversed-phase column (150 mm × 4.6 mm × 3 µm, 100 Å) purchased from Phenomenex (Torrance, CA, USA). The column temperature was set to 40 °C during the whole duration of the analysis. The mobile phase (Table 6) consisted of a gradient prepared from LC/MS-grade methanol and ultrapurified water prepared by the Simplicity Ultra-Pure Water Purification System (Merck Millipore, Billerica, MA, USA). LC/MS-grade formic acid was used as an organic modifier. Flow rate was maintained at 0.5 mL/min throughout the analysis. The total analysis time was 35 min.

Detection was carried out on a quadrupole rod mass spectrometer, with electrospray ionization (ESI), in both negative and positive multiple-reaction-monitoring (MRM) ion modes. Table 7 and Table 8 contain results obtained for the tested references. The temperature was set to 300 °C. Nitrogen was used for vaporization and as drying gas at 30 psi at 10 L/min. The capillary potential was set to +3000 V.

The injection volume was 1 μL for each reference and was maintained at each concentration. Identification was performed by comparison of the retention times, MS spectra, and the transitions between the separated compounds and references. Identification and quantification were performed based on the main transition from the MS spectra of each compound. Calibration curves were determined (R^2^ = 0.9964–0.9999) for the quantification of the compounds and references. The present method was validated by assessing linearity, precision, and accuracy according to International Conference on Harmonization (ICH) guidelines. LOD and LOQ were calculated after injecting a series of different concentrations for each analyzed sample. Extracts were evaluated for precision under optimized conditions. The method’s accuracy was determined in duplicate with a recovery experiment. All samples were injected in triplicate. Results obtained during validation can be found in Table 8, which presents calibration curve equations, correlation factors, and limits of detection (LOD) and quantification (LOQ).

### 4.6. Cell Culture

The cytotoxicity assay of L-R and L-DOX was performed using the SK-Hep-1 (HTB-52^™^) cell line derived from the ascitic fluid of a patient with adenocarcinoma of the liver [50] and the LX-2 (RRID: CVCL_5792) human hepatic stellate cell line [51]. The SK-Hep-1 (HTB-52^™^) cells were maintained in Minimum Essential Medium Eagle (MEM) supplemented with 10% fetal bovine serum and 1% Antibiotic-Antimycotic 100× and 1% L-glutamine in a 5% CO_2_ incubator (Advantage-Lab, Schilde, Belgium) at 37 °C in a humidified atmosphere, according to standard conditions. The LX-2 cell line was maintained in Dulbecco’s modified Eagle’s medium (DMEM, high glucose) supplemented with 10% fetal bovine serum and 1% Antibiotic-Antimycotic 100× and 1% L-glutamine [52,53].

### 4.7. Cytotoxicity Assay

The cytotoxicity of L-R and L-DOX was assessed using the (3-(4,5-dimethylthiazol-2-yl)-2,5-diphenyl tetrazolium bromide (MTT) assay according to a previously published protocol. Briefly, for each of the selected cell lines, 1 × 10^4^ cells/well were seeded on 96-well plates with 200 µL complete culture medium. After 24 h incubation, different concentrations of L-R (56.6 µM, 113 µM, 169.5 µM, 226 µM, and 282.5 µM), of doxorubicin (positive control) (1.64 µM, 8.2 µM, 12.3 µM, 16.4 µM, and 20.5 µM) and of the loaded liposomes were added. The concentrations were determined according to the amount of total polyphenols (TPC, µmol GAE/mL) in the L-R and L-DOX, respectively. Other controls were represented by untreated cells (negative control) and cells treated with L-E (internal control). Following 24 h incubation at 37 °C in a humidified atmosphere with 5% CO2, the medium was removed and 100 µL of 1 mg/mL MTT solution was added to determine the viability of the cells. After 3 h of incubation at 37 °C in dark, the MTT solution was removed from each well and 150 µL of dimethyl sulfoxide solution (DMSO) was added. Spectrophotometric readings at 450 nm were performed with a BioTek Synergy 2 microplate reader (Winooski, VT, USA). The cell viability percentages (%) were determined based on the absorbance ratio between cell cultures treated with L-R and L-DOX and the negative controls (untreated cells) multiplied by 100. The concentrations required to inhibit 50% of cell proliferation (IC_50_ values) for L-R and L-DOX were calculated from the dose–response curve obtained using non-linear regression. Each experiment was performed in triplicate [52,53,54].

### 4.8. Apoptosis Assay

Apoptosis was assessed with the Apoptosis & Necrosis Quantification Kit (Biotium, Fremont, CA, USA). Cells were stained with Annexin V-FITC and Ethidium Homodimer III (EthD-III) according to the kit’s instructions, and the fluorescent intensity was read with a BD FACS Canto II flow cytometer (Becton Dickinson, Franklin Lakes, NJ, USA) with a two-laser configuration: 20 mW argon solid state at 488 nm, and 17 mW neon-helium (NeHe) at 633 nm. The instrument was set to acquire information from the corresponding photodetector for Annexin V (FL1-A) and EthD-III (FL3-A) on a logarithmic scale. An unstained control sample was analyzed for FSC-A (forward scatter) and SSC-A (side scatter) signals in order to identify the cell population of interest and remove debris. The fluid pressure was set to a minimum (low) so the acquisition speed was appropriate. Subsequently, the stained samples were read. Fluorescence detection was achieved with the 488 nm laser and 525/50 filter for Annexin V and the 695/40 filter for EthD-III. A total of 10,000 events (cells) was run for each tube. Analysis was performed using the BD FACSDiva 6.1.2 software. Fluorescent intensity was shown in dot plots, divided into 4 quadrants each. Cells that did not bind any of the fluorochromes appeared in quadrant 3 (Q3—viable cells). Cells that were only stained with Annexin V-FITC appeared in quadrant 1 (Q1—apoptotic cells), whereas cells that were stained with both Annexin and EthD-III appeared in quadrant 2 (Q2—late apoptotic cells). Cells in quadrant 4 (Q4) were only stained with EthD-III and were necrotic cells. The percentages of apoptotic cells were calculated from Q2 and Q3. A total of 10,000 cells was analyzed and included in each dot plot.

### 4.9. Antimicrobial Activity Assays

To investigate the in vitro antimicrobial activity of the L-R, several methods were selected. The initial screening was based on the agar well-diffusion assay, a modified EUCAST (European Committee on Antimicrobial Susceptibility Testing) [55] disk-diffusion method.

Seven reference strains were included: *Staphylococcus aureus* ATCC 25923 (methicillin-susceptible *S. aureus*, MSSA), *Staphylococcus aureus* ATCC 700699 (methicillin-resistant *S. aureus*, MRSA), *Bacillus cereus* ATCC 14579, *Enterococcus faecalis* ATCC 29219, *Escherichia coli* ATCC 25922, *Salmonella enterica* serovar Typhimurium ATCC 14028, and *Salmonella enterica* serovar Enteritidis ATCC 13076. For each organism, an inoculum was made by suspending pure culture in Mueller Hinton (MH) broth for 24 h to obtain 10^−6^ colony-forming units (CFU)/mL according to the McFarland scale. The MH agar plate surface was “flood-inoculated” with the bacterial inoculum and prepared for extract evaluation; 6 m-diameter wells (three for each sample) were aseptically made in the MH agar to contain 60 μL of the tested product and 70% ethanol in water *v*/*v* (as the negative control). Gentamicin was also included as standard antibiotic. The growth-inhibition-zone diameters in millimeters were measured after 24 h of incubation at 37 °C. Minimum inhibitory (MIC) and bactericidal (MBC) concentrations were determined by a broth-microdilution method. Two-fold serial dilutions were made in 100 µL broth; 5.0 µL of a 24 h 10^−6^ CFU/mL bacterial inoculum were added to each well and incubated for 24 h at 37 °C. MIC values were read as the lowest concentrations able to inhibit the visible growth of bacteria (no turbidity in the well) compared to the negative control (broth). From each well, 10.0 µL were cultured on MH agar plates for 24 h at 37 °C. MBC values were read as the lowest concentrations associated with no visible bacterial growth on the agar plates. All these tests were performed in triplicate. Based on the MBC/MIC ratio, the MIC index was also calculated for each sample to evaluate whether the extract exhibited bactericidal (MBC/MIC ≤ 4) or bacteriostatic (MBC/MIC > 4) effect against the tested bacterial strains [56].

### 4.10. Statistical Analysis

All statistical analyses were performed using ANOVA GraphPad Prism software version 6.0 (GraphPad, San Diego, CA, USA). The obtained results are expressed as the mean ± standard deviation (SD). One-way analysis of variance (ANOVA) was used, followed by Tukey’s post hoc test, to determine statistical significance. A *p*-value lower than 0.05 was considered statistically significant [54].

## 5. Conclusions

*R. officinalis* is a widely known species with numerous therapeutic uses that are related to its polyphenols and essential oils. The present study provides novel and original insight into the species by proposing special delivery systems, liposomes encapsulating the leaf extract, for the evaluation of its antimicrobial and antiproliferative effects due to polyphenols. Not only were these biological activities confirmed, but novel insights on them and on their mechanism of action were also revealed. The results obtained therefore represent an important basis for further studies of innovative delivery systems loaded with different products of rosemary, which could be used for the treatment of numerous diseases as different pharmaceutical formulations. Moreover, the present study raises important arguments to offer a solid scientific background to sustain their use in the treatment of hepatic-related pathologies or antimicrobial ones. In this way, important perspectives for future studies on the species and its formulations are open in order to further elucidate mechanisms of action or to confirm the biological activity of the species.

## Figures and Tables

**Table 1:** Quality attributes of liposomes loaded with *R. officinalis* leaf extract.

Quality Attribute	Drug Content (μmol GAE/mL)	Encapsulation Efficiency (EE%)	Particle Size (nm)	Polydispersity Index (PdI)	Zeta Potential (mV)
Liposomes					
Empty liposomes (L-E)	-	-	308.9	0.207	−58.6
Liposomes loaded with DOX (L-DOX)	0.32	45.2	208.46	0.127	−4.67
Liposomes loaded with *R. officinalis* (L-R)	4.52	52.31	190.3	0.216	−26.5

**Table 2:** Results obtained for the LC-MS analysis of *R. officinalis*-loaded liposomes.

Compound	Retention Time (min)		*m/z* and Main Transition		Concentration (μg/mL)
	Reference	Separated Compound	Reference	Separated Compound	
Caffeic acid	13.8	13.6	179.0 > 135.0	179.0 > 135.0	81.07 ± 0.76
Chlorogenic acid	12.0	12.0	353.0 > 191.0	353.0 > 191.0	14.10 ± 0.12
Apigenin	28.2	28.1	269.0 > 117.0	269.0 > 117.0	2.26 ± 0.04
Chrysin	29.7	30.0	253.0 > 143.0	253.0 > 143.0	1.89 ± 0.02
Luteolin	26.9	26.8	287.0 > 153.0	287.0 > 153.0	0.85 ± 0.02
Luteolin-7-O-glucoside	19.9	19.8	447.0 > 284.9	447.0 > 284.9	7.42 ± 0.04
Quercetin	25.7	27.0	300.9 > 151.0	300.9 > 151.0	0.61 ± 0.02
Rutoside	20.3	20.3	609.0 > 300.0	609.0 > 300.0	9.86 ± 0.06
Naringenin	26.3	26.9	271.0 > 119.0	271.0 > 119.0	0.42 ± 0.02
Hesperetin	27.1	27.0	301.0 > 164.0	301.0 > 164.0	9.89 ± 0.12
Carnosic acid	32.0	32.0	331.2 > 285.1	331.2 > 285.1	20.03 ± 0.16
Rosmarinic acid	21.4	21.6	358.9 > 161.0	358.9 > 161.0	39.81 ± 0.35
Ellagic acid	27.3	27.3	301.0 > 185.0	301.0 > 185.0	880.02 ± 0.14
Carnosol	30.6	31.0	329.1 > 285.1	329.1 > 285.1	2.69 ± 0.04
Hyperoside	20.3	20.2	463.1 > 300.0	463.1 > 300.0	14.21 ± 0.18

**Table 3:** Results of the apoptosis assays for the *R. officinalis* extract-loaded liposomes.

Treatment	Viable Cells (%)	Apoptotic Cells (%)	Late Apoptotic Cells (%)	Necrotic Cells (%)
LX-2 L-E	94.2	0.0	1.5	4.2
LX-2 L-R-C1	87.5	10.3	1.8	0.4
LX-2 L-R-C2	94.8	3.8	1.0	0.4
LX-2 L-R-C3	96.9	1.9	0.9	0.3
LX-2 L-R-C4	95.9	2.5	1.3	0.3
LX-2 L-R-C5	94.8	3.6	1.3	0.4
LX-2 L-DOX-C1	74.7	11.0	13.6	0.8
LX-2 L-DOX-C2	72.4	10.8	15.2	1.6
LX-2 L-DOX-C3	70.4	15.8	12.2	1.5
LX-2 L-DOX-C4	67.5	12.9	17.5	2.1
LX-2 L-DOX-C5	75.0	9.1	13.2	2.7
SK-Hep-1 L-E	96.4	2.1	0.9	0.5
SK-Hep-1 L-R-C1	90.9	1.6	4.2	3.2
SK-Hep-1 L-R-C2	88.2	2.0	5.4	4.4
SK-Hep-1 L-R-C3	59.8	6.6	28.2	5.5
SK-Hep-1 L-R-C4	56.8	3.8	21.7	17.8
SK-Hep-1 L-R-C5	40.5	20.9	29.2	9.4
SK-Hep-1 L- DOX-C1	82.6	6.7	4.5	6.3
SK-Hep-1 L-DOX-C2	88.7	8.7	1.8	0.8
SK-Hep-1 L-DOX-C3	87.1	8.1	3.9	0.9
SK-Hep-1 L-DOX-C4	74.2	9.5	14.7	1.6
SK-Hep-1 L-DOX-C5	46.3	7.6	40.1	6.1

**Table 4:** In vitro antibacterial activity of L-R (agar well-diffusion assay).

	Zone of Inhibition (mm)	
Bacterial Species	L-R	Gentamicin
MSSA	25.33 ± 0.47	18 ± 0.00 ^a^
MRSA	20.67 ± 0.47	17 ± 0.00 ^b^
*Bacillus cereus*	27.33 ± 0.94	21 ± 0.00 ^b^
*Enterococcus faecalis*	18.33 ± 0.94	17 ± 0.00
*Salmonella enterica* serovar Enteriditis	16.33 ± 0.47	18 ± 0.00
*Salmonella enterica* serovar Typhimurium	16.33 ± 0.47	17 ± 0.00
*Escherichia coli*	17.33 ± 0.47	17 ± 0.00

Note: Values represent the mean ± standard deviations of three independent measurements. ^a,b^ Means with different subscript letters within a row are significantly different at *p* < 0.05.

**Table 5:** In vitro antibacterial activity of L-R (broth microdilution assay).

Bacterial Species	MIC Index MBC (μmol GAE/mL)/ MIC (μmol GAE/mL)
MSSA	2 0.28/0.14
MRSA	2 0.28/0.14
*Bacillus cereus*	1 0.14/0.14
*Enterococcus faecalis*	2 0.56/0.28
*Salmonella enterica* serovar Enteriditis	1 1.13/1.13
*Salmonella enterica* serovar Typhimurium	1 1.13/1.13
*Escherichia coli*	1 1.13/1.13

Note: Values represent the mean of three independent measurements.

**Table 6:** LC-MS mobile-phase gradient.

Time (min)	% Methanol	% Water	% of 2% Formic Acid in Water
0.00	5	90	5
3.00	15	70	15
6.00	15	70	15
9.00	21	58	21
13.00	21	58	21
18.00	30	41	29
22.00	30	41	29
26.00	50	0	50
29.00	50	0	50
29.01	5	90	5
35.00	5	90	5

**Table 7:** The LC-MS quantification parameters of references.

Reference	Retention Time (min)	*m/z* and Main Transition	MRM	Other Transitions
Caffeic acid	13.8	179.0 > 135.0	Negative	179.0 > 134.0 179.0 > 89.0
*trans-p*-coumaric acid	17.5	163.0 > 119.0	Negative	163.0 > 93.0
Chlorogenic acid	12.0	353.0 > 191.0	Negative	
Apigenin	28.2	269.0 > 117.0	Negative	
Chrysin	29.7	253.0 > 143.0	Negative	253.0 > 119.0 253.0 > 107.0
Luteolin	26.9	287.0 > 153.0	Positive	
Luteolin-*7-O*-glucosid	19.9	447.0 > 284.9	Negative	
Quercetin	25.7	300.9 > 151.0	Negative	300.9 > 121.0
Rutoside	20.3	609.0 > 300.0	Negative	609.0 > 301.0 609.0 > 271.0
Naringenin	26.3	271.0 > 119.0	Negative	271.0 > 107.0
Hesperetin	27.1	301.0 > 164.0	Negative	301.0 > 136.0 301.0 > 108.0
Carnosic acid	32.0	331.2 > 285.1	Negative	
Ellagic acid	27.2	301.0 > 185.0	Negative	301.0 > 257.0
Carnosol	30.7	329.1 > 285.1	Negative	
Kaempferol	28.0	285.0 > 187.0	Negative	285.0 > 151.0 285.0 > 133.0
Vitexin	18.4	431.0 > 311.0	Negative	
Rosmarinic acid	21.4	358.9 > 161.0	Negative	358.9 > 133.0
Myricetin	13.6	317.0 > 179.0	Negative	317.0 > 151.0 317.0 > 137.0
Hyperoside	20.3	463.1 > 300.0	Negative	463.1 > 301.0
Quercitrin	18.4	447.0 > 229.9	Negative	
Isoquercitrin	17.9	353.1 > 173.2	Negative	
Ferulic acid	18.4	193.0 > 134.0	Negative	193.0 > 178.0
Sinapic acid	18.4	223.0 > 207.9	Negative	
Gallic acid	7.0	168.9 > 125.0	Negative	

**Table 8:** LC/MS- The quantification parameters of the standards.

Reference	Concentration Range (mg/mL)	Calibration Curve Equation	Correlation Factor	Detection Limit (μg/mL)	Quantification Limit (μg/mL)
Caffeic acid	0.11–1.10	A = 4 × 10^7^ × C − 319,689	0.9998	3.20	4.80
*trans-p*-coumaric acid	0.16–1.60	A = 3 × 10^7^ × C + 291,065	0.9993	1.90	3.90
Chlorogenic acid	0.13–1.30	A = 2 × 10^8^ × C − 269,699	0.9997	5.00	8.00
Apigenin	0.10–0.98	A = 2 × 10^8^ × C + 15,916	0.9999	0.20	0.30
Chrysin	0.10–1.00	A = 1 × 10^8^ × C − 82,818	0.9997	3.00	5.00
Luteolin	0.01–0.10	A = 2 × 10^8^ × C − 2295.4	0.9977	0.05	0.07
Luteolin- *7-O*-glucosid	0.07–0.70	A = 1 × 10^9^ × C − 700,317	0.9990	3.00	4.00
Quercetin	0.09–0.91	A = 5 × 10^7^ × C − 9556	0.9964	0.80	1.10
Rutoside	0.17–1.70	A = 2 × 10^8^ × C − 191,937	0.9996	4.00	6.00
Naringenin	0.16–1.60	A = 3 × 10^8^ × C − 43,443	0.9999	0.60	0.90
Hesperetin	0.10–1.00	A = 6 × 10^7^ × C − 49,247	0.9974	3.00	5.00
Carnosic acid	0.28–2.80	A = 10^7^ × C − 99,360	0.9994	4.00	6.00
Ellagic acid	0.107–1.070	A = 14,987 × C − 138.52	0.9982	3.70	5.50
Carnosol	0.022–0.220	A = 10^9^ × C − 253,279	0.9997	1.00	2.00
Kaempferol	0.10–1.00	A = 10^7^ × C − 20,574	0.9996	0.80	1.20
Rosmarinic acid	0.028–0.278	A = 2 × 10^8^ × C − 6664.7	0.9996	0.10	0.20
Myricetin	0.140–1.400	A = 26,499 × C − 41.803	0.9997	0.60	0.90
Hyperoside	0.012–0.107	A = 4 × 10^8^ × C − 567,182	0.9986	0.60	0.90
Isoquercitrin	0.140–1.400	A = 4727 × C + 68.172	0.9973	2.90	5.80
Ferulic acid	0.100–1.000	A = 5 × 10^6^ × C − 50,000	0.9992	4.00	6.00
Gallic acid	0.107–1.070	A = 8 × 10^6^ × C − 37,131	0.9999	1.90	2.80

Note: A = peak area; C = concentration (mg/mL).

## Data Availability

Not applicable.

## Supplementary Materials

The following supporting information can be downloaded at: https://www.mdpi.com/article/10.3390/molecules27133988/s1, Figures S1. LC-MS chromatogram of *R. officinalis* loaded liposomes. Figure S2. Antimicrobial effect of the *R. officinalis* extract loaded liposomes by well diffusion method.
