# Supplementary material for: Antiproliferative and Antimicrobial Effects of Rosmarinus officinalis L. Loaded Liposomes
Source: Molecules. 2022 Jun 21;27(13):3988. doi: 10.3390/molecules27133988 (PMC9268459; doi:10.3390/molecules27133988)
Supplement: Supplementary file 1 [file molecules-27-03988-s001.zip › molecules-1769650-supplementary.pdf]

molecules

Supplementary Materials

Antiproliferative and Antimicrobial Effects of *Rosmarinus officinalis* L. Loaded Liposomes

Irina Ielciu [1], Mihaela Niculae [2], Emoke Pall [2], Cristina Barbălată [3,*], Ioan Tomuţă [3], Neli-Kinga Olah [4,5], Ramona Flavia Burtescu [4], Daniela Benedec [6], Ilioara Oniga [6] and Daniela Hanganu [6]

[1] Department of Pharmaceutical Botany, Faculty of Pharmacy, "Iuliu Haţieganu" University of Medicine and Pharmacy Cluj-Napoca, 400010, Romania; irina.ielciu@umfcluj.ro (I.I.)
[2] Department of Clinical Sciences, University of Agricultural Sciences and Veterinary Medicine Cluj-Napoca, 400374 Romania; mihaela.niculae@usamvcluj.ro (M.N.); emoke.pall@usamvcluj.ro
[3] Department of Pharmaceutical Technology and Biopharmaceutics, Faculty of Pharmacy, "Iuliu Haţieganu" University of Medicine and Pharmacy, Cluj-Napoca, 400010, Romania; barbalata.cristina@gmail.com (C.B.); tomutaioan@umfcluj.ro (I.T.)
[4] PlantExtrakt Ltd, Rădaia, 407059 Cluj-Napoca, Romania; neli.olah@plantextrakt.ro (N.K.O.); ramona.burtescu@plantextrakt.ro (R.B.)
[5] Department of Medicinal Chemistry and Pharmaceutical Industry, Faculty of Pharmacy, "Vasile Goldiş" Western University of Arad, 310414 Arad, Romania; olah.neli@uvvg.ro (N.O.)
[6] Department of Pharmacognosy, Faculty of Pharmacy, "Iuliu Haţieganu" University of Medicine and Pharmacy, Cluj-Napoca 400010, Romania; dbenedec@umfcluj.ro (D.B.); ioniga@umfcluj.ro (I.O.); dhanganu@umfcluj.ro (D.H.);

* Correspondence: barbalata.cristina@gmail.com (C.B.), irina.ielciu@umfcluj.ro (I.I.)

S. Supplementary Materials – Figures S1-S2

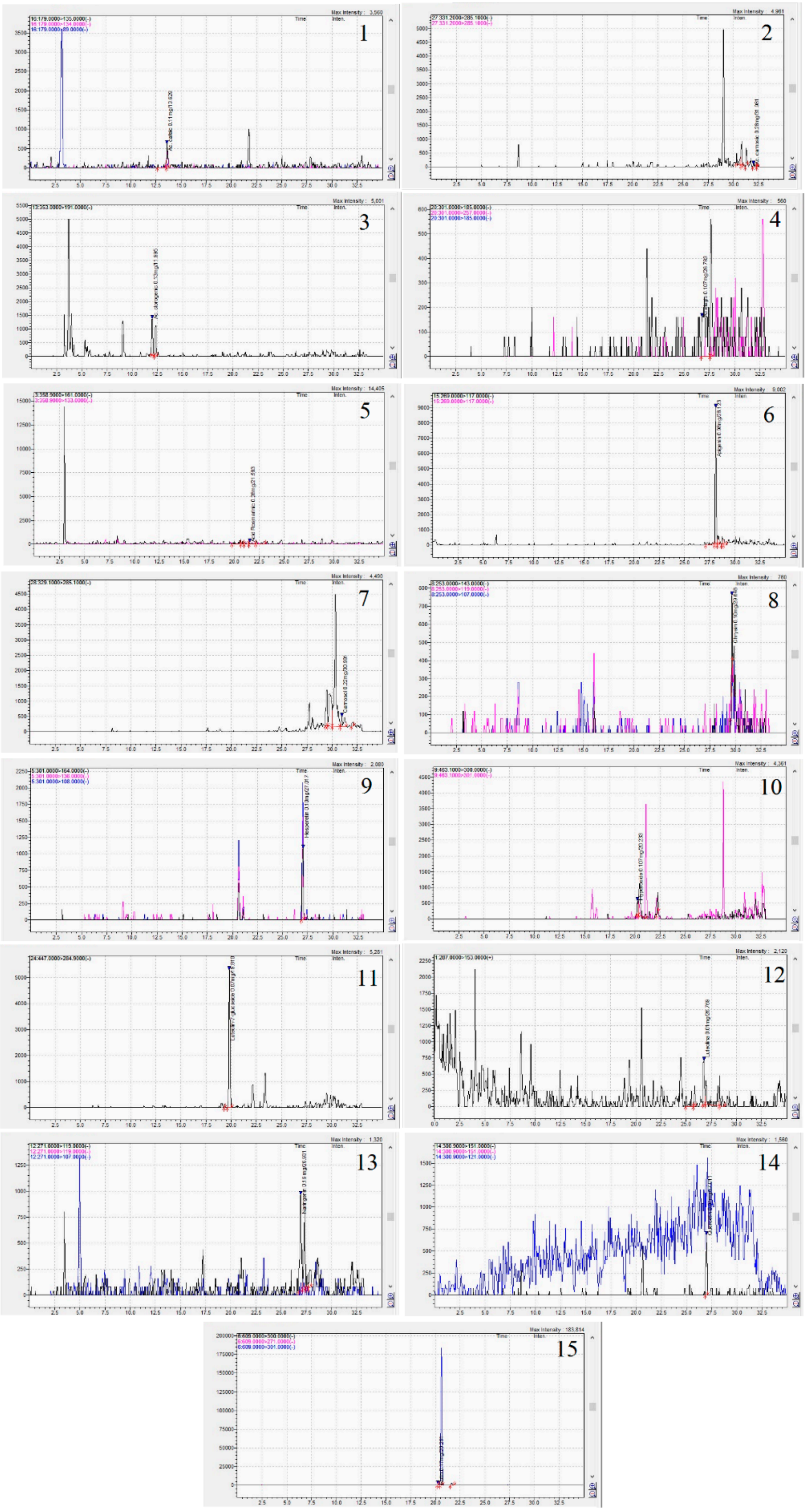

Figure S1. LC-MS chromatogram of *R. officinalis* loaded liposomes.

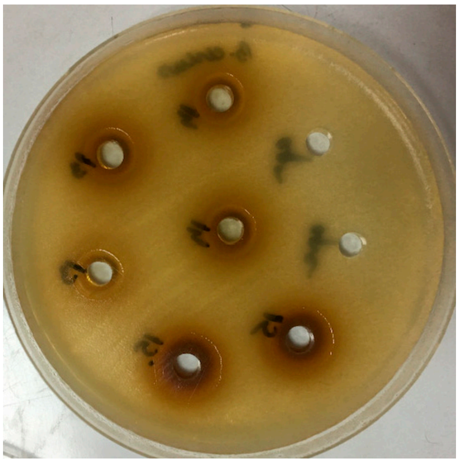

Figure S2. Antimicrobial effect of the *R. officinalis* extract loaded liposomes by well diffusion method.
